# Supplementary material for: Attachment Trauma Is Associated with White Matter Fiber Microstructural Alterations in Adolescents with Anorexia Nervosa before and after Exposure to Psychotherapeutic and Nutritional Treatment
Source: Brain Sci. 2023 May 14;13(5):798. doi: 10.3390/brainsci13050798 (PMC10216391; doi:10.3390/brainsci13050798)
Supplement: Supplementary file 1 [file brainsci-13-00798-s001.zip › brainsci-2297818-supplementary.pdf]

Supplemental Table S1. Cross-sectional and longitudinal analyses of MD.

Overlap of cluster region	kE	MNI coordinates			t value	*p*-value corrected at the cluster level (FWE)	Height Threshold
		x	y	z			
**Significant regional differences in MD values in the 21 AN patients compared to the 18 HC participants**							
**Significant MD increases in the 21 acute AN patients compared to the 18 HC participants at Tp1**							
Corpus callosum and the cingulum bilaterally extending to the corticospinal tract and the fornix	21,744	−4	−58	28	8.2	<0.001	0.001
Arcuate posterior segment bilaterally	510	58	−50	16	6	0.001	
Subcortical regions	497<br>492	−40<br>46	50<br>2	6<br>10	5.6<br>5.5	0.001	
**Significant MD decreases in the 17 AN patients from Tp1 to Tp2**							
Corpus callosum and the cingulum, bilaterally spreading to the corticospinal tract and the fornix, as well as subcortical regions	23818	38<br>4<br>−12	32<br>−32<br>−5	−12<br>18<br>16	9.2	<0.001	0.001

Supplemental Table S2. Correlations between FA values and CSF volume in the clinical group at baseline.

Overlap of cluster region	kE	MNI coordinates			t value	*p*-value corrected at the cluster level (FWE)	Height Threshold
		x	y	z			
**Significant correlations between FA values and CSF volume in the 21 acute AN patients at Tp1**							
Predominantly fornix, adjacent periventricular regions	366	−2<br>2	−8<br>−16	14<br>−4	6.9	<0.001	0.001
Inferior occipitofrontal and longitudinal fasciculus, left	191	−32	−52	−2	6.3	0.003	

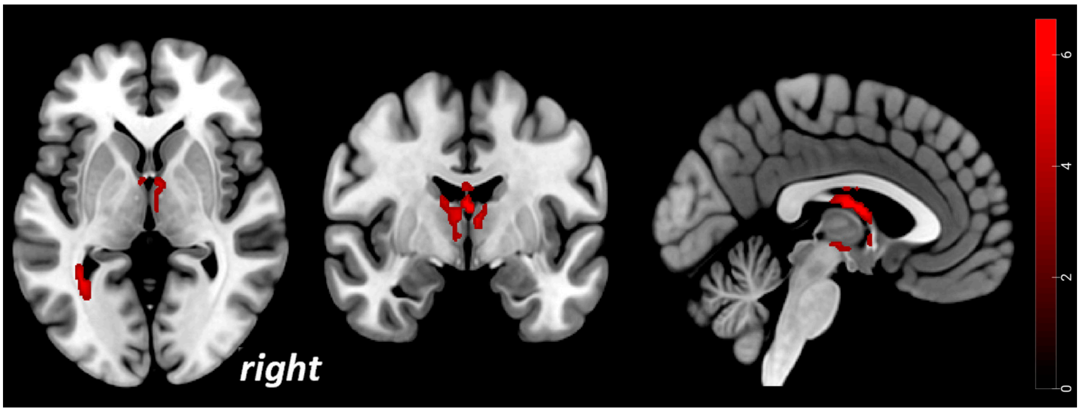

Supplemental Figure S1. Statistical parametric mapping (t) intensity projection maps rendered onto a stereotactically normalized MRI scan: voxel cluster of the significant correlations between whole CSF volume and FA alterations in the 21 acute AN patients (statistical significance is thresholded at $p < 0.001$,

FWE $p < 0.05$ corrected at the cluster level). The right side of the image corresponds to the right side of the brain.
